# Application of IgG-Derived Natural Treg Epitopes (IgG Tregitopes) to Antigen-Specific Tolerance Induction in a Murine Model of Type 1 Diabetes

**DOI:** 10.1155/2013/621693

**Published:** 2013-04-23

**Authors:** Leslie P. Cousens, Yan Su, Elizabeth McClaine, Xin Li, Frances Terry, Robert Smith, Jinhee Lee, William Martin, David W. Scott, Anne S. De Groot

**Affiliations:** ^1^EpiVax, Inc., 146 Clifford Street, Providence, RI 02903, USA; ^2^University of Maryland School of Medicine, Baltimore, MD 21201, USA; ^3^Alpert Medical School of Brown University, Providence, RI 02912, USA; ^4^University of Massachusetts Medical School, Worcester, MA 01655, USA; ^5^Uniformed Services University of Health Sciences (USUHS), Bethesda, MD 20814, USA

## Abstract

HLA class II-restricted regulatory T cell (Treg) epitopes in IgG (also called “Tregitopes”) have been reported to suppress immune responses to coadministered antigens by stimulating the expansion of natural Tregs (nTregs). Here we evaluate their impact on human immune responses to islet cell antigens *ex vivo* and on the modulation of type 1 diabetes (T1D) in a murine model *in vivo*. Co-administration of Tregitopes and T1D antigens delayed development of hyperglycemia and reduced the incidence of diabetes in NOD mice. Suppression of diabetes could be observed even following onset of disease. To measure the impact of Tregitope treatment on T cell responses, we evaluated the effect of Tregitope treatment in DO11.10 mice. Upregulation of FoxP3 in KJ1-26-stained OVA-specific CD4^+^ T cells was observed following treatment of DO11.10 mice with Tregitopes, along with reductions in anti-OVA Ig and T effector responses. In *ex vivo* studies of human T cells, peripheral blood mononuclear cells' (PBMC) responses to GAD65 epitopes in the presence and absence of Tregitope were variable. Suppression of immune responses to GAD65 epitopes *ex vivo* by Tregitope appeared to be more effective in assays using PBMC from a newly diagnosed diabetic subject than for other more established diabetic subjects, and correlation of the degree of suppression with predicted HLA restriction of the Tregitopes was confirmed. Implementation of these defined regulatory T cell epitopes for therapy of T1D and other autoimmune diseases may lead to a paradigm shift in disease management.

## 1. Introduction

Induction of antigen-specific tolerance is a logical strategy for immunological therapy for type 1 diabetes (T1D). T1D is an organ-specific autoimmune disease resulting from the destruction of insulin-producing pancreatic islet beta cells. In nondiabetics, islet antigen-specific T cells are deleted during thymic development, rendered anergic, or converted to regulatory T cells (Tregs) that actively suppress effector responses to islet cell antigens. In persons with T1D and in the non-obese diabetic (NOD) mouse model of T1D, these tolerance-inducing mechanisms do not function properly. In humans, defects in Tregs have been proposed as one mechanism by which individuals develop T1D, and this defect is said to be both functional and quantitative [1]. In the absence of effective regulatory suppression, CD8^+^ and CD4^+^ auto-reactive T cells respond to islet antigens presented by human leukocyte antigen (HLA) molecules. The gradual destruction of islet cells by these auto-reactive cells eventually leads to glucose intolerance.

Natural Tregs (nTregs) are an important component of immune regulation in the peripheral circulation, suppressing auto-reactive T cell responses to unrelated antigens by both contact-dependent and -independent mechanisms [2]. Expansion of CD4^+^CD25^hi^FoxP3^+^ nTregs is being considered as a potential novel therapy for treatment of diabetes [3]. Ideally, as described here, administration of nTregs or nTreg-inducing therapies in conjunction with islet cell “target antigen(s)” would convert auto-reactive T cells to adaptive Tregs, restoring antigen-specific tolerance. Expansion of nTregs is generally performed through non-specific means (with IL-2) since, with few exceptions [4, 5], the antigen specificity of nTregs is still unknown. Autoimmune responses can be modulated by nTregs by inhibiting the antigen-specific activity of nearby auto-reactive effectors and/or by changing the phenotype of the effectors to an induced Treg (iTreg) phenotype. 

**Figure 1:**
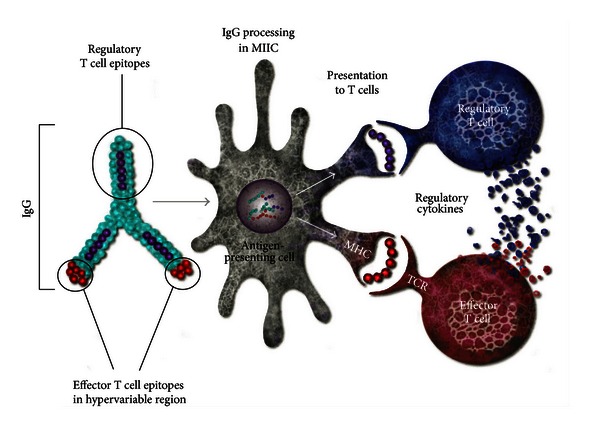
Postulated Tregitope mechanism of action. Tregitopes are highly conserved Treg epitopes found in human and other species' IgG. They are postulated to reduce the immunogenicity of neo-epitopes in the hypervariable region of IgG CDR; they also suppress immune response to other co-delivered T cell epitopes. (A version of this figure was originally published in Blood. De Groot A. S., Moise L., McMurry J. A., Wambre E., Van Overvelt L., Moingeon P., Scott W., Martin W. Activation of natural regulatory T cells by IgG Fc-derived peptide “Tregitopes”. Blood 2008; 112 : 3303.)

We previously identified a set of Treg epitopes derived from immunoglobulin G (IgG) that induces Tregs to expand and leads to antigen-specific tolerance [6] (Figure 1). We hypothesized that these Tregitopes (T regulatory cell epitopes) are natural T cell epitopes contained in IgG; their presence in the highly conserved domains of IgG may explain why intravenous immunoglobulin therapy is associated with the expansion of nTregs in mice and in humans [7, 8]. Tregitopes can be defined as peptides that (a) bind to multiple MHC class II molecules, (b) induce Tregs to suppress effector T cell responses to co-delivered antigen, and (c) upregulate Treg-associated cytokines and chemokines. T cells that expand in response to Tregitopes exhibit a T regulatory phenotype (CD4^+^CD25^hi^FoxP3^+^) [6]. In addition to the Tregitopes that were first described in 2008, several Tregitopes that fit the above criteria have been identified in the Fab (framework) and Fc regions of IgG. We and others have suggested that Tregitopes may occur in other common serum proteins such as albumin [9]. The Tregitopes used in the present study include two previously described human Tregitopes (hTregitopes), 167 and 289, and two murine homologs of hTregitopes 167 and 289 (mTregitopes), which are located in the CH1 and CH2 domains of IgG Fc, respectively.

Tregs are known to suppress T cell responses directly and indirectly [10]. In concordance with existing theories about nTregs, direct suppression of bystander T cells may be mediated by Tregitope-specific nTregs [10, 11] through expression of certain cytokines and/or by modulation of the antigen-presenting cell (APC) towards a tolerance-inducing phenotype. Indirect suppression (not specific to the T cell epitope or Tregitope recognized by the nTreg) would be mediated by adaptive Tregs, also known as iTregs [2, 10]. In the present study, we tested the ability of Tregitopes to suppress immune responses *in vivo* with and without murine preproinsulin (PPI, islet cell antigen) epitopes in the NOD model of T1D. We also treated DO11.10 mice with Tregitopes, so as to determine the effect on bystander effector cells *in vivo*, and utilized well-defined reagents to examine antigen-specific T cells from these mice. The DO11.10 studies permitted an examination of Tregitope effects elicited solely due to induction of nTregs and the subsequent induction of iTregs that, in the case of DO11.10, would be OVA antigen specific.

If, as demonstrated here, activation of nTregs by Tregitopes and the subsequent induction of iTregs could slow or halt the immune destruction of islet beta cells in the pancreas, Tregitopes might be used early in diabetes as a treatment for preserving endogenous insulin production. Moreover, the potential for Tregitopes to induce antigen-specific tolerance (as compared to more broadly immunosuppressive treatments) could contribute to a shift in clinical management of autoimmune disease, away from immunosuppression and towards antigen-specific immunomodulation. 

## 2. Materials and Methods

### 2.1. *In Silico* Immunoinformatics Methods

#### 2.1.1. EpiMatrix

EpiMatrix is a T cell epitope-mapping algorithm that is used to identify putative HLA ligands/T cell epitopes, such as Tregitopes, contained within protein sequences (e.g., epitopes identified from pathogen genomes in references [12, 13]). Computation is performed by comparing peptide sequences with a set of HLA allele-specific coefficient matrices. To complete an analysis using the EpiMatrix algorithm, target protein sequences are parsed into overlapping 9-mer frames in which each frame overlaps the last by eight amino acids. Each amino acid in the 9-mer is then assigned a positive or negative coefficient based on its previously determined propensity to positively or negatively impact peptide binding when located at that amino-acid position within the HLA-binding groove [14]. The coefficients are then summed to produce a raw score for each 9-mer. Raw scores are normalized with respect to a distribution derived from a large set of randomly generated peptide sequences. The resulting Z-scores from this distribution are directly comparable across predictions for different alleles.

#### 2.1.2. ClustiMer

By performing this operation repeatedly on a large range of protein sequences, from pathogens to autologous proteins, we have determined that T cell epitopes are not randomly distributed throughout protein sequences but instead tend to “cluster” together within a protein sequence [15]. T cell epitope “clusters” range from 12 to roughly 25 amino acids in length and can contain anywhere from four to 40 binding motifs in the overlapping 9-mer frames. ClustiMer preferentially identifies epitope clusters restricted by multiple HLA class II alleles covering 95% of human populations [16]; clusters selected in this manner demonstrate strong HLA binding affinity and T cell responses [13, 17–19]. Many of the most immunogenic T cell epitope clusters contain a feature we now refer to as an EpiBar [20]. An EpiBar is a single 9-mer frame that is predicted to be reactive to at least four different HLA alleles. Peptides containing EpiBars appear to have a greater-than-expected ability to induce T cell responses of either a stimulatory or suppressive phenotype. This may be due to the co-location of strong HLA binding motifs [12, 13]. 

#### 2.1.3. Selection of GAD65 Epitopes and Clusters

**Figure 2:**
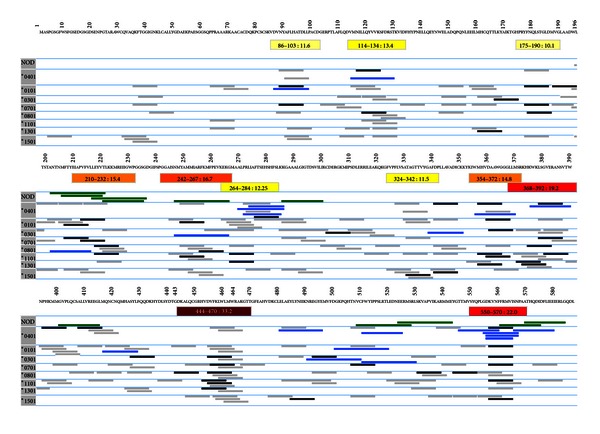
Map of GAD65 epitopes performed using EpiMatrix. EpiMatrix-predicted epitopes are shown in shades of grey and black; darker colors denote stronger binding potential. Published human epitopes are in bright blue. Clusters are identified in shades of yellow-orange-red; again, stronger colors (more red) denote higher predicted immunogenicity. Previously published NOD epitopes are shown in green. The clustering of predicted (and published) epitopes is clearly illustrated for EpiMatrix-predicted epitope clusters 450–470 (shown in greater detail in Figure 3(a)) and 550–570 (Figure 3(b)).

Based on EpiMatrix analysis of GAD65, we selected 14 peptides containing EpiBars for *in vitro* HLA binding assays and T cell assays using peripheral blood mononuclear cells (PBMC) from T1D subjects. The human GAD65 sequence was obtained from the GenBank sequence database at the National Center for Biotechnology Information (NCBI: Accession Q05329). This sequence was parsed into 577 overlapping 9-mers and scored for predicted HLA binding affinity using the EpiMatrix algorithm as previously described [21, 22].  Figure 2 illustrates the locations of GAD65 clusters (highlighted in shades of red that correspond with overall EpiMatrix score) selected by EpiMatrix, as well as a comparison between EpiMatrix predictions and T1D epitopes published in a recent compendium (in blue [23]). Details of the sequences tested *in vitro* are also provided in greater detail in Table 1.

**Figure 3:**
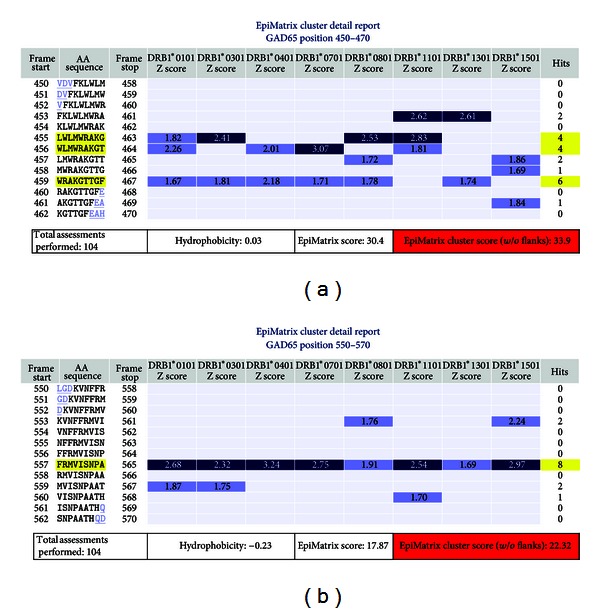
GAD65 450–470 and 550–570 cluster maps. EpiMatrix-predicted 9-mer hits for eight prevalent HLA class II alleles are shown in the cluster map under each allele for two example GAD 65 peptide sequences. Any 9-mer peptide scoring above 1.64 on the EpiMatrix “Z” scale (top 5%) is considered to be a potential epitope (blue bars) or “hit.” Peptides scoring above 2.32 on the scale (top 1%) are extremely likely to bind MHC (dark blue bars). The 450–470 cluster contains three 9-mers with multiple positive scores. Hits across four or more HLA class II alleles constitute an EpiBar. The GAD65 450–470 cluster contains three EpiBars; the 550–570 cluster contains a single EpiBar (highlighted in yellow).

Figures 3(a) and 3(b) provide more detailed EpiMatrix cluster reports for two GAD65 peptides. “EpiBars” are present in these peptides. For the cluster located at amino acid position 450 (GAD65 450, Figure 3(a)), one EpiBar begins at Frame 455, a second starts at 456, and a third (weaker due to lower overall scores) at 459. This GAD65 sequence has not previously been identified or published as an epitope. As illustrated in Figures 2 and 3(b), there are published GAD65 epitopes located in peptide GAD65 550 at frame 557; the same sequence contains an EpiBar.

#### 2.1.4. iTEM Analysis

The iTEM (Individualized T cell Epitope Measure) tool provides an estimate as to whether a given individual will develop a T cell response to a protein antigen, based on HLA binding predictions. We use a mathematical formula that converts DRB1 allele binding predictions generated by EpiMatrix into an allele-specific scoring system. Thus, iTEM can be used to define an HLA binding threshold, above which immune response is likely to be present and below which immune response is likely to be absent for individual study subjects. iTEM scores have been shown to correlate with immune responses to peptides in T cell assays *in vitro* [24]. An iTEM score was calculated for each of the Tregitope peptides, and the result was compared to *in vitro* responses generated in the two-step ELISpot assay.

### 2.2. Peptides and Peptide Synthesis

All peptides used in this study were synthesized by 9-fluoronylmethoxy-carbonyl (Fmoc) synthesis to a purity of >80% as determined by HPLC by New England Peptide (Gardner, MA, USA) and 21st Century Biochemicals (Marlborough, MA, USA). The peptide masses were confirmed using either a Q-Star nanospray Mass Spectrometer (New England Peptide) or by MALDI-TOF mass spectrometry (21st Century Biochemicals). Third party QC was performed by Cell Essentials (Cambridge, MA, USA). Tregitope peptides are as previously described [6]; murine Tregitopes 167 and 289 are as described in Elyaman et al. [25]. Tregitope sequences, murine PPI peptide sequences, and class II-binding control peptide sequences are listed in Table 2. Murine Tregitopes 167 and 289 each contain a single significant score (1.98 and 1.73, resp.) for the murine class II MHC allele I-Ag7 (NOD) based on *in silico* analysis using an EpiMatrix experimental predictive matrix. Irrelevant control peptide TetTox_831–845_ contained slightly lower predicted binding scores for the NOD MHC allele (maximum score of 1.51). 

### 2.3. Liposome Preparation

Sterically stable cationic liposomes were prepared from three lipid components: dioleylphosphatidylethanolamine (DOPE), dimethylaminoethane-carbamoyl-cholesterol (DC-cholesterol), and polyethylene glycol 2000-phosphatidyl-ethanolamine (PEG). The lipids were mixed, dried in a rotary evaporator, and resuspended in phosphate-buffered saline (PBS; Sigma-Aldrich, St. Louis, MO, USA) to make empty multi-lamellar vesicles. These vesicles were sonicated five times for 30 seconds each at 4°C to convert them into unilamellar liposomes. Unilamellar liposomes (10 nmol) were mixed with peptides, flash frozen, and freeze-dried overnight. To encapsulate peptides in liposomes, the resulting powder was resuspended with sterile distilled water and vortexed for 15 seconds every five minutes for 30 minutes at room temperature. PBS was added to yield a final liposome concentration of 10 mM lipid/mg peptides. Vesicles <150 nm in diameter were produced by 20–30 cycles of extrusion through polycarbonate filters using a Liposofast extruder (Avestin, Canada). Liposome formulations were stored at 4°C until use.

### 2.4. *In Vivo* Study Methods

#### 2.4.1. Mouse Models

Non-obese diabetic (NOD/ShiLtJ) mice, a polygenic model for T1D, were purchased from the Jackson Laboratory (Bar Harbor, ME, USA). NOD/ShiLtJ mice exhibit diabetes beginning at around 12 weeks of age, characterized by insulitis, a leukocytic infiltrate of the pancreatic islets [26]. Plasma glucose levels are used to determine disease onset. T cell receptor (TCR) transgenic DO11.10/FoxP3-GFP and DO11.10/FoxP3-GFP/Rag2^−/−^ were developed as described in [27]. Briefly, a FoxP3-GFP mouse was crossed against a line of either DO11.10 or DO11.10/Rag2^−/−^ mice and backcrossed to create homozygous DO11.10/FoxP3-GFP lines. All animals were housed and bred in pathogen-free micro-isolator cages at the animal facilities operated by the University of Maryland School of Medicine. 

#### 2.4.2. Immunizations

Groups of female mice were immunized with either peptides or protein in one of several delivery vehicles: PBS, incomplete Freund's adjuvant (IFA; Sigma-Aldrich), or liposomes (described above). Immunizations were given as indicated for each of the experiments, either intraperitoneally or in one hind footpad by needlestick. Additional experimental details are provided in the section devoted to each of the assays. 

#### 2.4.3. T Cell Phenotyping and Proliferation Assays (DO11.10)

Draining inguinal and popliteal nodes were harvested and the samples were evaluated by FACS for T cell proliferation and Treg expansion. The KJ1-26 mAb that reacts with the clonotypic TCR expressed by DO11.10 transgenic mice was used to identify T cells expressing the transgenic OVA-specific TCR. To detect FoxP3/GFP^+^ Tregs, a single-cell suspension of draining lymph nodes was incubated with 2.4G2 mAb (anti-CD16/32, ATCC) for 15 minutes to block FcR, then stained with CD4-PE and anti-clonotypic KJ1-26-APC for 40 minutes at 4°C. Cells were acquired on a FACSCalibur flow cytometer (Becton-Dickinson, Franklin Lakes, NJ, USA) and analyzed using FlowJo software (Treestar, Ashland, OR, USA). The CD4^+^KJ1-26^+^ live cell gate population was established, and these gated cells were analyzed for the presence of GFP fluorescence indicative of FoxP3 expression. 

Antigen-specific T cell proliferation was evaluated by [^3^H] thymidine incorporation [27]. Draining lymph nodes were harvested and a single-cell suspension was prepared at 2 × 10^6^ cells/mL. Cells were added to 96-well plates at 100 *μ*L per well in the presence of indicated concentration of OVA 323–339 (New England Peptide, Gardner, MA, USA). Forty-eight hours later, the cells were pulsed with 1 *μ*Ci/well of [^3^H] thymidine and incubated for another 16–20 hours. Cells were then harvested on glass fiber filters, and [^3^H] incorporation into the DNA was measured using a MicroBeta2 plate counter. 

### 2.5. *In Vitro* Study Methods

#### 2.5.1. HLA Binding Assays

Of the 16 GAD65 peptides selected for this study, 14 could be synthesized and purified to ≥80% pure; GAD65 324-342 was not included in the studies reported here due to technical difficulties in peptide synthesis, and GAD65 108-131 was not included due to low purity. Therefore, we validated 14 epitopes in HLA-DR binding studies for use in *ex vivo* two-step ELISpot assays with human PBMC. 

Human Tregitopes and GAD65 epitopes were evaluated for binding to HLA *in vitro*. We used an HLA binding assay initially described by Steere et al. [28] and adapted for higher throughput in our laboratory. In 96-well plates, a test peptide and a reference peptide competed for binding to purified class II HLA molecules (provided by William Kwok, Benaroya Institute, Seattle, WA, USA) for 24 hours at 37°C. The nonbiotinylated test peptides were evaluated over a range of concentrations, while the biotinylated reference peptide was held at a fixed concentration (0.1–1 *μ*M depending on the HLA). The reference peptides and concentrations were as follows: biotin-Flu-HA 306–318 (PRYVKQNTLKLAT) at 0.1 *μ*M for DRB1*0101 and DRB1*0401, and at 1 *μ*M for DRB1*0701; and biotin-MBP 84–102 (NPVVHFFKNIVTPRTPPPS) at 0.5 *μ*M for DRB1*1501. The peptides that bound to class II molecules were then captured on ELISA plates using pan anti-class II antibodies (L243, anti-HLA-DR alpha chain). The assays were developed by addition of streptavidin-europium and read on a time-resolved fluorescence plate reader. Standard curve analysis using four-parameter logistics was performed in SigmaPlot software, and an IC_50_ value was calculated for each peptide-allele pair in which 50% inhibition was encompassed by the range of test peptide dilutions (Table 1). Some peptides were available in limited supply and thus were only tested for binding to DRB1*0401.

#### 2.5.2. Study Subjects

Subjects diagnosed with T1D and who possessed the HLA-DR4 allele were recruited at the Hallett Center for Diabetes and Endocrinology, Rhode Island Hospital (Providence, RI, USA). Negative control subjects were recruited under a separate IRB protocol in collaboration with Dr. William Beliveau of Clinical Partners LLC (Johnston, RI, USA). These human studies were conducted in conformance with local institutional review board guidelines.

#### 2.5.3. Human PBMC Two-Step T Cell Assays

PBMC from individual subjects were isolated from whole blood using Ficoll gradient separation. The buffy coat was washed in PBS and resuspended in RPMI containing 10% human AB serum (Valley Biomedical, Winchester, VA, USA), 1% L-glutamine (Sigma-Aldrich) and 0.1% gentamycin (Sigma-Aldrich). All cells were prestimulated with a pool of up to 14 peptides derived from GAD65 at 2.5 *μ*g/mL per peptide as well as exogenous IL-2 (10 IU/mL) and IL-7 (20 ng/mL), and half were also treated with 5 *μ*g/mL each of human Tregitopes 167 and 289. The cells were plated at a density of 2.5 × 10^6^ viable cells/mL and cultured for 7 days in 24-well culture plates containing 2 mL PBMC suspension in each well. Every 2-3 days during this period, half of the medium was replaced with fresh medium containing IL-2 and IL-7. At the end of the primary incubation (step 1), the cells were harvested and restimulated in ELISpot plates (step 2) with individual GAD65 epitopes, the peptide pool, or whole GAD65 protein (Kronus, UK).

#### 2.5.4. ELISpot Analysis (Human PBMC)

The frequency of epitope-specific T lymphocytes was determined using Mabtech IFN*γ* ELISpot plates (Mabtech, Sweden). After the initial expansion in culture (step 1), cells were washed three times, transferred to ELISpot plates at 200,000–250,000 cells per well, and incubated at 37°C for 48 hours with individual peptides, peptide pools, or whole GAD65 protein in triplicate (step 2). A negative control (no stimulus) and mitogen control (PHA) were included. Spot counts were determined by a third-party vendor (Zellnet Consulting, Inc., Fort Lee, NJ, USA) using a Zeiss ELISpot plate reader (Table 3). Responses were considered to be positive if two criteria were satisfied: (1) the average number of spots in the triplicate test wells was at least twice the average of the background wells, and (2) the average number of spots was at least 50 spots per 1 million cells over background. Due to variability in amounts of blood obtained from study subjects, cell yields following separation of PBMC from whole blood, and recovery after the primary culture, not all subject PBMC samples were tested with every GAD65 peptide in the ELISpot assays, as indicated by “ND.” Reduced spot counts in Tregitope-treated cultures, as summarized in Table 4, were tested for statistical significance, relative to the corresponding spot counts generated by cells not treated with Tregitopes, by a one-tailed Student's *t*-test performed on the pair of ELISpot triplicates. 

## 3. Results

### 3.1. *In Vivo* Study Results

#### 3.1.1. Tregitope Treatment of NOD Mice Prior to Onset of Diabetes

The objective of the *in vivo* studies was to determine whether Tregitopes co-administered with or without mPPI peptides could prevent the onset of diabetes in the well-characterized NOD murine model of spontaneous autoimmune diabetes. In the *first prevention study*, we dosed female NOD mice three times intraperitoneally (i.p.) at 9, 10, and 11 weeks of age (female; 10 mice/group, 3 groups total) with either (1) an irrelevant OVA peptide (control peptide, 100 *μ*g per animal) delivered in saline, (2) saline control, or (3) mTregitope 289 and mTregitope 167 in saline (50 *μ*g per Tregitope peptide, 100 *μ*g total per animal). Blood glucose (BG) levels were measured twice weekly for up to 30 weeks; the mice were considered to be diabetic if their BG level was ≥250 mg/dL for two consecutive measurements.

**Figure 4:**
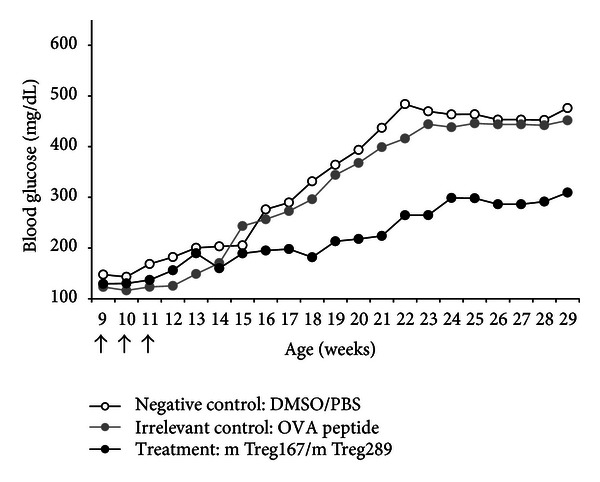
Study 1—prevention of T1D in NOD mice using Tregitopes. Tregitopes in saline, given IP prior to onset of diabetes, had a moderately suppressive effect on development of diabetes in NOD mice. The incidence of diabetes for each arm is shown along the course of the study beginning at the first week that the mice received treatment. Arrows represent time points when Tregitope treatment was administered. Mice receiving vehicle alone (white circles) or irrelevant peptide (gray circles) had a higher incidence of diabetes at 29 weeks of age than mice receiving Tregitope treatment (black circles).

Treatment with Tregitopes alone (without the addition of islet cell antigen peptides) administered i.p. at 9, 10, and 11 weeks of age had a moderately suppressive effect on the development of diabetes in NOD mice (Figure 4). Among all 3 groups of mice tested in this experiment, BG levels began to increase around 11 to 13 weeks of age; some mice became diabetic at weeks 11-12. Administration of the two Tregitopes (mTregitope 167 and mTregitope 289) reduced the incidence of diabetes and average BG levels as compared to the two control groups (OVA peptide and saline treatment); this effect became apparent starting at weeks 16-17 and lasted for the duration of the study (to week 29). These results were not statistically significant although trends were evident and consistent with follow-up studies, as discussed below.

In *the second prevention study,* NOD mice (female; 8 mice/group) were divided into five treatment groups (A–E), each of which received five injections (at the onset of disease in weeks 8–10, and again in weeks 14-15) of their respective treatment. In this study, we administered Tregitopes with the T1D-associated murine preproinsulin (mPPI) epitopes to determine whether Tregitopes alone or Tregitopes in conjunction with target antigen led to a better outcome.

*Group A* (mPPI) received the five murine PPI peptides listed in Table 2 in liposomes; *Group B* (mPPI + Tregitopes) received mPPI peptides plus four Tregitope peptides (h167, h289, m167, and m289) in liposomes; *Group C* (mPPI + Tet Tox) received mPPI peptides with a control peptide (MHC-binding tetanus toxin peptide 830–844) in liposomes; *Group D* (Tregitopes) received four Tregitope peptides alone in liposomes; and *Group E* (Liposomes) received empty liposomes. Tregitopes were dosed at 50 *μ*g per mouse per Tregitope peptide, for a total of 200 *μ*g per mouse. mPPI peptides were dosed at 10 *μ*g/mouse per peptide, for a total of 50 *μ*g per mouse. Tet Tox was administered at 200 *μ*g per mouse. BG was measured as described above. All treatments were started prior to onset of disease (weeks 8–10) and continued for a total of five treatments. 

**Figure 5:**
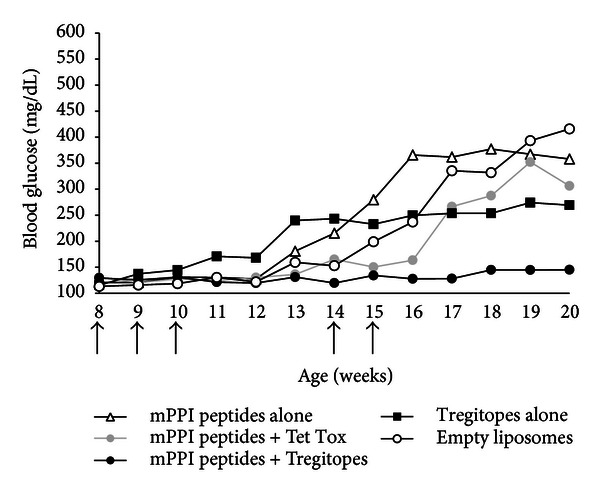
Study 2—prevention of T1D in NOD mice using Tregitopes. When co-administered with the “target” antigen (five murine preproinsulin or mPPI peptides) in liposomes, treatment with four Tregitope peptides (mouse 167 and 289 and human 167 and 289) completely suppressed the development of T1D in NOD mice. Arrows represent time points when treatments were administered. Black symbols represent groups receiving Tregitopes with (circle) or without (square) target antigen. White circles represent the vehicle control group. Antigen peptides alone are indicated by white triangles, and antigen peptides with an irrelevant MHC-binding peptide by gray circles. Mice receiving liposomes alone (white circles) had a 67% incidence of diabetes at 20 weeks of age compared to 38% in mice receiving Tregitopes alone (black square). None of the mice receiving Tregitopes with antigen (black circles) developed diabetes.

Treatment with mPPI and mouse and human Tregitopes 167 and 289 suppressed the development of T1D in NOD mice (Figure 5). None of the mice treated with Tregitope + mPPI peptides (Group B) developed diabetes, although half (50%) of the mice treated with mPPI peptides alone (Group A) became diabetic.

The suppression of diabetes incidence by Tregitopes was long lasting. At 20 weeks, mice treated with Tregitopes + mPPI (Group B) remained diabetes-free and showed no significant glucose elevation (*P* < 0.01). Two mice in Group D (Tregitopes) developed diabetes nearly two weeks before any other group, but by the end of the study had a lower overall incidence and lower levels of BG than any other group except for Group B. The three groups that did not receive any Tregitope treatment (Groups A, C, and E) all had an incidence of diabetes above 50% at 20 weeks and average BG measurements above 350 mg/dL.

#### 3.1.2. Tregitope Treatment of NOD Mice following Onset of Disease

The objective of the remaining two studies was to determine whether Tregitopes administered at the onset of diabetes could resolve hyperglycemia in the NOD model. Female NOD mice were monitored for BG daily and entered into treatment groups when BG levels were between 200–250 mg/dL on two successive days. Upon entry into the study, mice received a single injection of mTregitopes 167 and 289 (total 20 *μ*g) formulated in IFA in the flank by subcutaneous injection. Control mice received PBS in IFA (as a vehicle control) or were left untreated. BG levels were measured weekly following the treatment up to 30 weeks, and mice were considered to be diabetic if their BG level was ≥ 250 mg/dL. The experiment was conducted twice.

**Figure 6:**
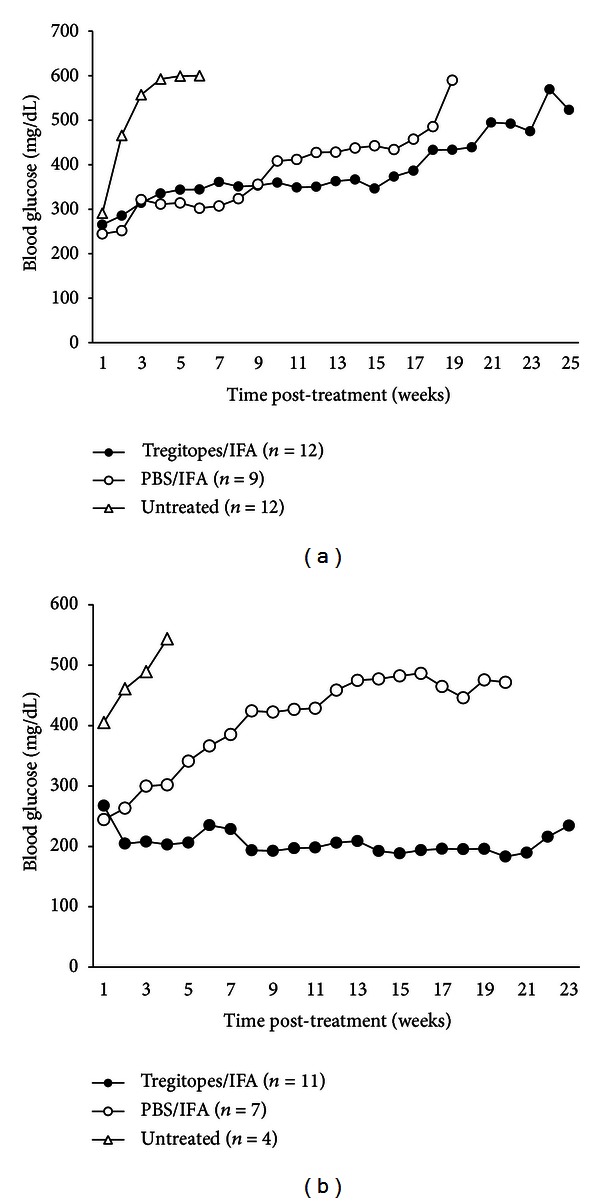
Treatment with Tregitopes in IFA at onset of diabetes. Female NOD mice were monitored for blood glucose (BG) levels. Disease onset was defined as BG between 200 and 250 mg/dL measured on two consecutive days, at which time mice were entered into the study. Mice received a single subcutaneous injection in the flank of either mTregitopes 167 and 289 (total 20 *μ*g) formulated in IFA or PBS in IFA (as a vehicle control) or they were left untreated. BG levels were then measured weekly. In the initial study (a), Tregitopes suppressed the progression of diabetes in seven of twelve mice, whereas IFA alone had less of an effect. This study was repeated (b), showing successful “cure” of diabetes after onset. All pair-wise comparisons are significant (*P* ≤ 0.05).

As shown in Figure 6(a), a single Tregitope-IFA treatment reduced BG over a period of 25 weeks in the first of these experiments. PBS-IFA controls exhibited initial suppression, but an eventual rise of BG was observed in the majority of mice. The group that received Tregitopes in IFA had a stronger and longer-lasting effect. By 25 weeks on study (approximately 35 weeks of age), seven of twelve (58%) Tregitope-treated diabetic mice had reduced BG from greater than 250 to normal levels and remained non-diabetic, whereas only two of nine (22%) PBS controls remained non-diabetic, and all twelve (100%) untreated mice had developed diabetes within two weeks. When the percentages of diabetes-free mice in each group were plotted over time as Kaplan-Meier curves and analyzed using the log-rank test and pairwise Holm-Sidak comparisons, there was a significant (*P* ≤ 0.005) difference between the untreated and Tregitope-treated groups, but not between the PBS-IFA and Tregitope-treated groups.

In the second experiment (Figure 6(b)) all untreated control mice rapidly progressed, reaching BG of 500 mg/dL within 2-3 weeks. In the group of mice that received Tregitope-IFA, ten of eleven (91%) mice had BG less than 250 mg/dL at 26 weeks on study (approximately 36 weeks of age), and four (36%) of these had transient elevations that self-corrected, suggesting that there was a long-lasting effect of the Tregitope treatment on immune homeostasis. In general, mice for which treatment was initiated when their BG was greater than 350 mg/dL did not respond to Tregitope treatment. In the PBS-IFA group, three of seven (43%) mice had BG lower than 250 mg/dL at the end of 20 weeks on study (30 weeks of age), suggesting that IFA alone may have a partial effect on the development of diabetes. Nonetheless, there were significant (*P* ≤ 0.05) differences in diabetes-free percentages over time between the Tregitope-treated group and both untreated and PBS-IFA-treated groups by the Holm-Sidak method.

If the two experiments are combined, seventeen of 23 (74%) of the initially diabetic mice in the Tregitope-IFA group had BG lower than 250 mg/dL at the end of observation. In contrast, only five of sixteen (31%) mice treated with vehicle alone (PBS in IFA) had a BG level lower than 250 mg/dL at the end of 20 weeks, suggesting Tregitopes delivered by depot injection (in IFA) at the onset of disease are able to reverse new-onset diabetes in NOD mice. 

#### 3.1.3. Antigen-Specific Adaptive Tolerance Induction

Antigen specificity could not be measured in the NOD model as reagents (antibodies staining TCR specific for islet cell antigens and tetramers for NOD mouse epitopes) were not available at the time these studies were performed. Instead, DO11.10 mice, a TCR transgenic (Tg) line in which the TCRs are specific for a peptide derived from the ovalbumin antigen (OVA), were employed to further examine effects of Tregitope on the induction of adaptive tolerance *in vivo*. In these mice, approximately 80% of CD4^+^ cells, of which 3–5% are naturally FoxP3^+^, possess a Tg TCR that is specific for OVA 323–339 peptide and recognized by the anti-clonotype mAb KJ1-26. The other 20% of CD4^+^ T cells, of which 10–15% are FoxP3^+^, have endogenously recombined TCRs. 

Six DO11.10 FoxP3/GFP knock-in Tg mice (generated from mice purchased from Jackson Laboratories as described in methods and in [27]) were each injected with 15 *μ*g of mTregitopes 167 and 289; five control DO11.10 FoxP3/GFP knock-in Tg mice were injected with an equivalent amount of MHC-binding irrelevant control peptide from influenza hemagglutinin (Table 2, Flu HA 306–318), emulsified in IFA via one hind footpad. After 10 days, mice were sacrificed and draining inguinal and popliteal lymph nodes were removed for T cell isolation. We measured (i) suppression of OVA-specific proliferative T cell responses to stimulation with OVA 323–339 peptide *ex vivo *by [^3^H] thymidine incorporation assay and (ii) induced GFP (FoxP3) expression in the CD4^+^/KJ1-26^+^ T cells by FACS after staining. 

**Figure 7:**
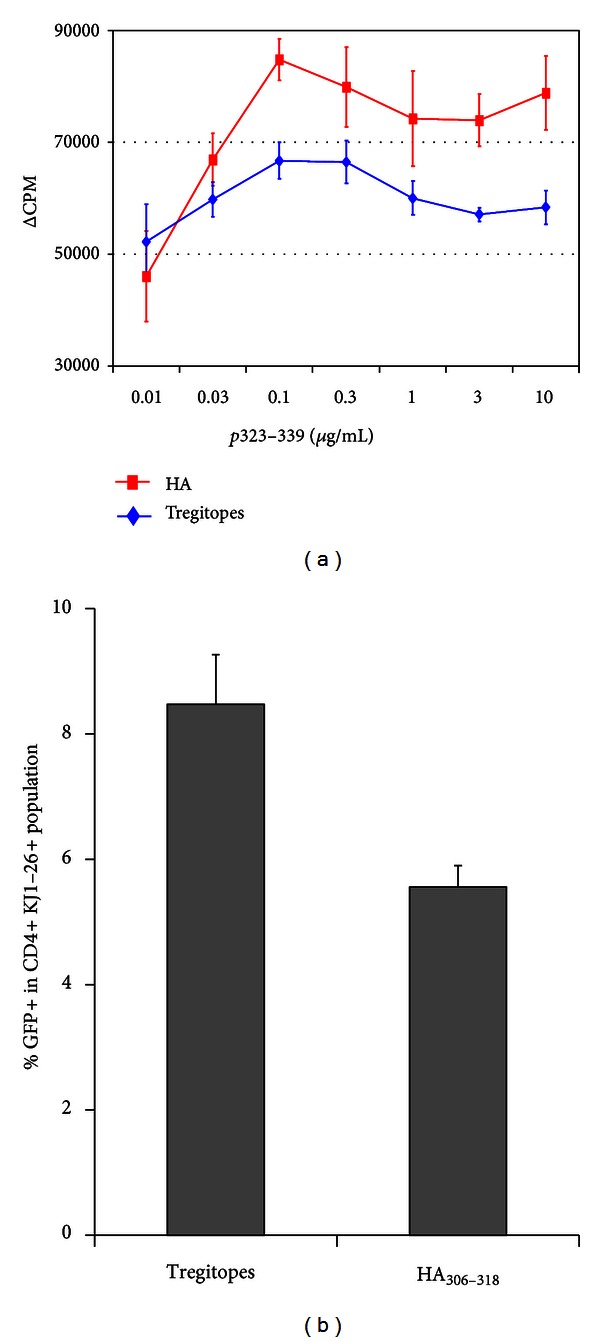
Tregitope effect in TCR-transgenic mice. DO11.10 FoxP3/GFP transgenic mice were immunized with mT167/mT289/IFA and an irrelevant peptide HA (influenza hemagglutinin, Table 2) in IFA. After 10 days, mice were sacrificed and assayed for (a) T cell proliferation by *in vitro* culture with pOVA, and (b) FoxP3/GFP (Tregs) expression by FACS after CD4 and KJ1-26 staining. Gamma interferon expression in cell-culture supernatants was also suppressed (data not shown).

As shown in Figure 7, Tregitope treatment suppressed OVA-specific proliferative T cell responses to OVA 323–339 peptide as compared to the control (Flu HA) and increased proportions of FoxP3-expressing CD4^+^ T cells bearing the OVA-specific Tg TCR (KJ1-26^+^). Of the CD4^+^ Tg T cells, more than 8% were FoxP3^+^ (*P* < 0.02 as compared to Flu HA control). This significant increase in FoxP3-GFP^+^/KJ1-26^+^ cells demonstrates adaptive tolerance induction, as the Tregitopes presented in murine MHC cannot be recognized directly by the OVA-peptide specific Tg T cells. A similar experiment was performed in Rag^−/−^ DO11.10 mice, which have OVA-specific T cells but lack endogenous TCR and therefore would lack Tregitope-specific nTregs. No suppression of *ex vivo* proliferation or IFN*γ* production was observed in this context (data not shown), suggesting that Tregitope-specific nTregs are required for Tregitope treatment-associated iTreg induction.

### 3.2. *In Vitro* Study Results: Epitope Prediction and Validation

#### 3.2.1. GAD65 Epitope Prediction

We performed a detailed *in silico* analysis of the islet cell antigen GAD65. As has been determined in other proteins, GAD65 T cell epitopes identified by EpiMatrix are not randomly distributed throughout the protein sequence but instead tend to “cluster” in specific regions, which overlap with previously published epitopes (Figure 2 and Table 1). The T cell epitope clusters range from twelve to roughly 25 amino acids in length and contain up to eight binding motifs for the eight common HLA alleles; this observation is consistent with previous descriptions of these epitopes as potentially highly reactive T cell epitope clusters for human T1D subjects and nondiabetic subjects [23, 29]. Sixteen epitope clusters in human GAD65 with strong binding motifs for DR4 and for at least four other HLA were identified. 

#### 3.2.2. HLA Binding Assays

Fourteen of the sixteen peptides identified by EpiMatrix and ClustiMer were synthesized to a purity of >80% and tested for binding affinity to DR4 by an in vitro competition-based HLA binding assay as described in Methods. In addition to performing HLA-DR4 binding studies, we evaluated binding to a number of selected HLA types. Among the fourteen epitopes predicted and synthesized, twelve were predicted to bind HLA-DR4. In the binding assay for this allele, seven showed high affinity (IC_50_ < 25 *μ*M) while another two showed weak affinity (IC_50_ > 50 *μ*M), for a total of nine true positive predictions. The remaining five peptides showed no DR4 affinity; two of these five non-binders had EpiMatrix scores below the threshold of significance, making them true negative predictions. Thus, EpiMatrix accurately predicted the binding assay results for eleven (79%) of the fourteen peptides. In our experience, lack of binding can be observed for longer sequences which may allow peptides to fold or aggregate *in vitro*, interfering with HLA binding.

Additional HLA binding assays were performed to define specific IC_50_ curves for binding to DRB1*0101, DRB1*0701, and DRB1*1501 (Table 1). Overall, when all EpiMatrix predictions (not just published HLA binding restrictions) are included, the peptides bound as predicted for more than 90% of cases. EpiMatrix predicted and confirmed several new HLA restrictions for the GAD65 DR4-restricted published epitopes. For example, for peptide GAD65 243–267, affinity for HLA-DR4 and numerous other alleles was predicted by EpiMatrix and experimentally validated in binding assays. GAD65 450–470 (Figure 3(a)), an unpublished epitope, also bound promiscuously (as predicted). 

#### 3.2.3. *Ex Vivo* Assays with Human PBMC

We evaluated human immune responses to the GAD65 peptides in the presence or absence of Tregitopes. Subjects are described in the methods section above. PBMCs from six DR4-positive subjects and five non-diabetic subjects (Table 3) were examined for interferon gamma (IFN*γ*) secretion in response to restimulation with individual GAD65 peptides after being cultured with a pool of those peptides in the presence or absence of hTregitope 167 and 289 by a two-step ELISpot assay (described in Methods). 

As shown in Table 3, seven of eleven subjects (64%) demonstrated broad baseline immune responses to GAD65 (with a number of spot-forming cells, or SFC, higher than 50 per 10^6^ cells over background to at least half of the epitopes). This is consistent with previously published results [23]. The average number of GAD65 epitopes for which positive IFN*γ* ELISpot responses were observed per T1D subject was 8.3 (Table 3). The average number of GAD65 epitopes for which positive IFN*γ* ELISpot responses were observed per normal (non-diabetic) control subject was not significantly different at 6.0 (Table 3). Thus, there were no clear differences observed in terms of IFN*γ*-secreting cell numbers and epitope-specific responses to GAD65 between diabetic and non-diabetic groups, as has been previously observed [23]. 

All fourteen tested peptides derived from GAD65 and selected using EpiMatrix induced significant IFN*γ* responses as measured by ELISpot in at least one of the tested subjects, confirming the predictions. Of note, the EpiMatrix-selected epitope GAD65 450–470 described in Figure 3 induced IFN*γ* responses in PBMC from five of six (83%) of the diabetic subjects and three of five (60%) negative control subjects; immune responses to this peptide have not been previously described in the literature. 

#### 3.2.4. Summary of Two-Step Assay Results

Following co-incubation of GAD65 peptides with Tregitopes 167 and 289 with PBMC *in vitro*, there was a trend towards a reduction in the recall response to the GAD65 peptides, although the inhibition levels varied significantly between patients and even between peptides tested for a given patient. This variation can be attributed to differences in the HLA affinity of the GAD65 epitopes and the Tregitopes for an individual subject's HLA alleles (see iTEM analysis, Section 3.2.4). In terms of ELISpot counts, as summarized in Table 4, Tregitope-treated PBMC mounted a diminished IFN*γ* response to 78% (diabetic subjects) or 74% (normal control subjects) of GAD65 epitopes included in each assay, as compared to PBMC not pre-cultured with Tregitopes. Reductions in spot counts were statistically significant (*P* < 0.05) for 26% of the peptides tested.

#### 3.2.5. Individualized T Cell Epitope Measure (iTEM) and T1D Responses

T cell responses to epitopes, whether T effector or T regulatory, may vary depending on HLA binding affinity, an effect that was previously described for Tregitopes [6]. We have developed a method for predicting whether an immune response (T effector or T regulatory) will be measured for a given subject, based on the predicted binding affinity of the study peptide for the subject's HLA alleles, called the individualized T cell epitope measure or iTEM [24]. Diabetic subjects' IFN*γ* ELISpot responses to GAD65 peptides were significantly associated with iTEM scores for each peptide by standard Chi-squared analysis (*P* < 0.005), whereas no statistically significant relationship was identified between iTEM scores and SFC for healthy control subjects (*P* > 0.35). To determine if the effect of Tregitopes was correlated with the iTEM score of the Tregitopes, iTEM scores were calculated for the Tregitopes in the 42 peptide/subject pairs for which the ELISpot showed a significant response to GAD65 epitope restimulation (greater than 50 SFC/10^6^ cells over background) in the absence of Tregitopes. When the degree of Tregitope suppression for the corresponding assay, performed with Tregitopes, was evaluated by linear regression analysis for the 42 peptide/subject pairs, no trend was observed between higher iTEM scores for the Tregitope peptide and the strength of the Tregitope effect. However, regression analysis of the 16 cases where a statistically significant suppression of the GAD65 immune response by Tregitope was identified indicated a greater correlation (*P* < 0.01) between the Tregitope iTEM score and the strength of the response to Tregitope (as measured by the actual decrease in the number of SFC/10^6^ cells). Specifically, higher iTEM scores for these 16 peptide/patient combinations were correlated with larger decreases in SFC/10^6^ cells following Tregitope co-incubation. The accuracy of the linear model, as indicated by the *R* ^2^ value, was greater for this data subset (*R* ^2^ = 0.64) than for all the ELISpot results (*R* ^2^ = 0.07). Thus the effect of individual Tregitopes based on HLA affinity for the DR alleles of individual subjects was reaffirmed in the present study [6].

#### 3.2.6. Relationship between Tregitope Efficacy and Time from Diagnosis

**Figure 8:**
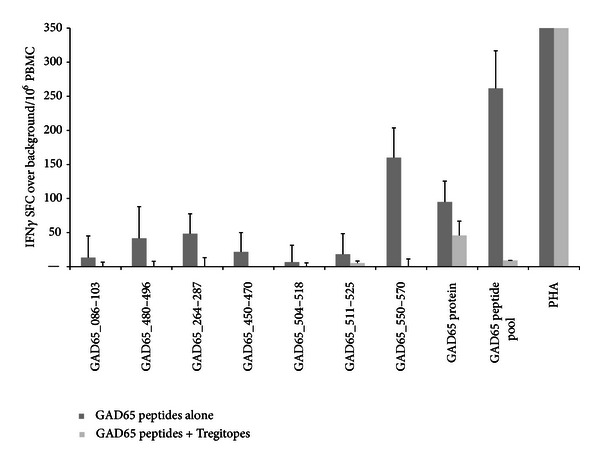
ELISpot results from a recently diagnosed T1D patient. Dark bars represent cells preincubated with a pool of GAD65-derived epitopes, and light bars represent cells precultured with the same pool plus Tregitope peptides. IFN*γ* ELISpot responses to GAD65 peptides incubated with PBMCs from a recently diagnosed subject (<6 months) were either low or did not meet criteria for a positive response. Tregitopes were able to suppress responses to the whole GAD65 protein and to the GAD65 peptides, individually or pooled.

We reasoned that Tregitope effects might be more pronounced early in the development of T1D, as the inflammatory responses to T1D antigens might be restricted to fewer epitopes. Taking a closer look at two recently diagnosed subjects, we found that subject 1107, diagnosed 7 months prior to the blood draw, had fewer responses to GAD65 epitopes, and those T cell responses that were present were lower and more easily suppressed by coincubation with Tregitope than in the other subjects. The onset of diabetes is difficult to determine with precision; thus the strong baseline responses and lack of Tregitope-mediated suppression in subject 1104 (Figure 8), who was diagnosed 6 months prior to blood draw, may be an indicator that the subject had diabetes for a substantial period of time before being medically diagnosed.

## 4. Discussion

### 4.1. *In Vivo* Studies

Novel therapies inducing long-lasting islet-specific tolerance and protection are needed for T1D. The critical period for intervention is at the onset of diabetes, when Tregs can protect islet cell mass [30]. In the studies described here, we demonstrated that co-administration of Tregitopes and mPPI peptides prior to onset of diabetes led to the abrogation of diabetes over a period of 15 to 20 weeks. Diabetes was completely suppressed to the end of the study (20 weeks) in mice treated with target antigens (mPPI) and Tregitope; a moderate effect was observed for Tregitope alone, and no effect was observed when mice were treated with mPPI peptides and control peptide Tet Tox or with empty liposomes. 

In a therapeutic model also using NOD mice dosed with mTregitopes 167 and 289 (and no additional target antigens), we show that the administration of Tregitopes in IFA following onset of diabetes led to a significant reduction in the incidence of diabetes as compared to saline control. In a repeat of this experiment, the duration of tolerance lasted longer than 15 weeks after only a single treatment with Tregitopes.

#### 4.1.1. Induction of Adaptive Tolerance

One potential explanation for the effect of Tregitopes in the NOD model would be induction of islet-cell antigen-specific tolerance. To test this, we evaluated the effect of Tregitope on antigen-specific adaptive tolerance in an existing TCR-Tg mouse model. TCR-Tg FoxP3-expressing iTregs (GFP-fluorescent in this model) were identified following treatment with mTregitopes 167 and 289 by gating on KJ1-26^+^ T cells (a marker for the Tg TCR). Treatment with the control peptide Flu HA 306–318 did not result in similar levels of iTregs. We also showed that these Tregs were functional, in that OVA peptide-specific proliferative T cell responses were suppressed, as was cytokine expression. In a repeat experiment performed in Rag^−/−^ DO11.10 mice (lacking nTregs), no Tregitope effect was observed, suggesting that the suppression of immune responses may require induction and activation of Tregitope-specific cells, which subsequently lead to modification of the phenotype of the TCR Tg T cells. Thus, the study was entirely consistent with a two-step model for adaptive tolerance induction by Tregitopes: (i) induction of Tregitope-specific KJ1-26^−^ (non TCR-Tg) nTregs followed by (ii) induction of KJ1-26^+^(TCR Tg) iTregs. In mechanism of action studies (Cousens et al., submitted for publication), we have confirmed that incubation of Tregitope with T cells and APC leads to the following chronological series of events: Treg expansion and activation, followed by modification of the APC phenotype (at 72 hours), which is in turn followed by induction of antigen-specific adaptive tolerance.

Adaptive tolerance to islet cell antigens is believed to be a key component to successful T1D therapy. The adaptive Treg induction specific to the OVA peptide, following treatment with Tregitopes, in DO11.10/FoxP3-GFP/TCR-Tg mice was similar to the amount of iTreg induction observed to a birch pollen antigen in our original published studies [6]. Moreover, van der Marel et al. delivered Tregitope 167 using an AAV in a mouse model of colitis and observed significant reductions in disease endpoints [31]. Protection from disease was associated with thymic Treg populations that increased from 7% in control- to 11% in Tregitope-treated mice. This provides independent evidence that even small increases in Treg populations can have a significant effect on the amelioration of inflammatory disease. Although Tregs were initially thought to originate exclusively in the thymus [32], subsequent studies in mice and humans showed that CD4^+^CD25^hi^FoxP3^+^ adaptive or induced Tregs can develop in the periphery under a variety of conditions [2, 33]. 

### 4.2. *In Vitro* Studies

#### 4.2.1. GAD65 Epitopes

Wicker et al. performed HLA DR4 binding assays using overlapping peptides from GAD65 and established that three high-binding peptides also stimulated proliferative T cell responses in Tg mice expressing HLA-DRB1*0401 [34]. A subsequent paper by Endl et al. confirmed that peptides 266–285 and 556–575 were T cell epitopes in diabetic subjects [35]; we confirmed these findings using HLA DR4 analysis by EpiMatrix [36]. The three sequences contain three of the top four GAD65 peptides selected by EpiMatrix. Additional published GAD65 epitopes can be found within the EpiMatrix-predicted clusters defined here. We describe one new epitope located at GAD65 amino acid 450 that has not been previously published. All of the published epitopes were revalidated in HLA binding assays and T cell assays, and one previously undiscovered epitope was identified in this study, confirming the utility of epitope-prediction tools such as EpiMatrix and ClustiMer for use in studies of autoimmunity. We found that type 1-diabetic and normal control subject PBMC responded similarly to T1D-associated antigens, as has been noted previously [23].

#### 4.2.2. Two-Step Assays

When we co-incubated human Tregitopes with PBMC *in vitro*, we observed many instances in which Tregitopes suppressed immune responses to epitopes derived from GAD65. GAD65 epitope responses differed by subject (as might be expected given HLA background), and the effect of the Tregitopes also varied by the baseline response to the GAD65 epitopes. In cases where the T cell response was stronger (more ELISpot-forming cells per million PBMC) the Tregitopes generally did not suppress immune responses. However, we did observe an overall correlation with the iTEM score for the Tregitopes and the Tregitope suppression *in vitro*. Although limited in terms of the number of subjects and the number of immune responses observed, the correlation between iTEM score (ability of the Tregitopes to be presented in the context of the subjects' HLA DR alleles) and suppression of immune response *in vitro *is consistent with previously published [6] and unpublished data (Federico Mingozzi, personal communication). 

Negative control wells contained medium alone; control peptides were not used in these *in vitro* studies. Immune responses seen in the subjects ranged from high to low for each of the peptides. Nonetheless, in previously published studies [6] and in studies submitted for publication (Cousens, mechanism of action studies), there is no comparable suppressive effect of control (MHC-binding) peptide *in vivo* or *in vitro*, as compared to Tregitopes, which have also been shown to directly induce the expansion of Tregs. Tregitope suppression was not due to toxicity, again, since global immunosuppression was not seen and the *in vitro* suppressive responses were highly correlated with the ability of the HLA to present the peptide as defined by iTEM. In addition, no toxicities were associated with Tregitope treatment in any of the four NOD studies described here. 

## 5. Conclusions

Reducing islet cell destruction and preserving islet cell function are the goals of therapies intended to cure T1D. Proposed treatments with anti-CD3 monoclonal antibodies (mAbs) such as Teplizumab, which induce Tregs, have shown some efficacy, but the mechanism of Treg induction is elusive and the effect appears to be either brief and/or requires treatment very early in T1D disease [37]. The critical period for intervention is at the onset of diabetes, when Tregs can protect islet cell mass [30]. Treg function may also be better preserved early in T1D. Similar drugs targeting effector T cells such as Abatacept and Otelixizumab may also be of limited efficacy, in part due to their lack of specificity for pathogenic T cells. Tregitopes administered with T1D antigens may induce antigen-specific tolerance and, for that reason, could be more effective than previously studied tolerance-inducing approaches. 

Tregitopes administered to NOD mice at onset of diabetes resolved hyperglycemia for as long as 29 weeks, and, in parallel studies with DO11.10 Tg mice, Tregitope treatment induced antigen-specific Tregs in these mice. Effective tolerance induction was achieved with a single dose of Tregitopes in IFA, and, taken together, the *in vivo* studies suggest that Tregitopes induce antigen-specific tolerance and that Tregitopes might be effective for the initial (early) treatment of T1D. Maintenance of tolerance might require intermittent low-dose IL-2 to maintain Treg populations [38].

Tregitope therapy may be considered similar to, but also distinct from, IVIG (polyclonal intravenous human immunoglobulin G) therapy [39]. In a number of autoimmune disease conditions in animals and in humans, IVIG has been shown to induce the expansion of Tregs and IL-10 secretion [40–43]; dendritic cell processing is required for the effect of IVIG in some studies [44]. Tregitopes are also similar to a number of other IgG-derived peptides (independently identified as Tregitopes due to their fulfillment of the standard Tregitope criteria) such as CDR1, also known as Edratide [45–47], which provides additional independent confirmation of Tregitopes. By inference from these human studies, Tregitope peptides administered to humans may have a safety profile similar to IVIG. 

Two additional peptide immunotherapies that resemble Tregitope in that they stimulate antigen-specific immunomodulatory T cell responses, GAD and DiaPep277, are in clinical trials. Each of these is based on a single peptide or protein. Unlike DiaPep, the Tregitope therapeutic strategy might include multiple Tregitope peptides from IgG administered in conjunction with islet cell antigens, which would enhance Treg responses to those antigens. Similarly, GAD immunotherapy of T1D patients might be enhanced by the addition of Tregitopes. 

And finally, initial studies of Tregitope in murine transplant studies demonstrate that they may be effective against chronic but not acute graft rejection (Nader Najafian, personal communication). Thus Tregitopes may also be of use in the context of regenerative approaches to treating T1D by increasing beta-cell mass and facilitating islet cell transplantation. Applications to other autoimmune diseases and enzyme-replacement therapy are also being considered [48, 49]. Whether as part of a preventive, therapeutic, or transplant regimen, induction of tolerance to islet cell antigens using Tregitopes could result in a paradigm shift in disease management, allowing clinicians to safely unlock the potential of Tregs to regulate immune responses and expand the number of immunotherapeutic strategies available to treat autoimmune diabetes.

## Figures and Tables

**Table 1:** GAD65 sequences and HLA binding IC_50_. Sequences for GAD65 epitopes were predicted using EpiMatrix (for high cluster score and if possible for high predicted HLA DR4 binding affinity) and synthesized for testing in HLA binding assays. The calculated IC_50_ values (*μ*M) for four HLA alleles are provided. “NB” indicates the peptide was a non-binder. The peptides were predicted and confirmed to be promiscuous binders (see Figure 2).

Peptide Name	Sequence	EpiMatrix Cluster Score	DRB1*0101 IC_50_	DRB1*0401 IC_50_	DRB1*0701 IC_50_	DRB1*1501 IC_50_	Published?
GAD65_086–103	DVNYAFLHATDLLPACDG	12.19	<5	7.5	<5	NB	Y (NOT DR4)
GAD65_175–190	HPRYFNQLSTGLDMVG	9.98	10.7	>50	13.9	NB	N
GAD65_203–226	NMFTYEIAPVFVLLEYVTLKKMRE	14.70	—	<25	—	—	Y (NOT DR4)
GAD65_243–267	GAISNMYAMMIARFKMFPEVKEKGM	15.87	<5	NB	8.7	<5	Y (NOT DR4)
GAD65_264–287	EKGMAALPRLIAFTSEHSHFSLKK	13.77	—	<25	—	—	Y
GAD65_338–352	TTVYGAFDPLLAVAD	9.69	4.1	NB	NB	31.3	Y (NOT DR4)
GAD65_354–372	CKKYKIWMHVDAAWGGGLL	14.48	NB	6.4	<5	>50	Y
GAD65_369–392	GGLLMSRKHKWKLSGVERANSVTW	17.60	16.3	<5	<5	NB	Y
GAD65_405–424	SALLVREEGLMQNCNQMHAS	4.45	NB	>50	NB	NB	Y (NOT DR4)
GAD65_450–470	VDVFKLWLMWRAKGTTGFEAH	33.90	25.4	NB	10.2	11.4	N
GAD65_480–496	YLYNIIKNREGYEMVFD	5.90	<5	5.7	NB	<5	Y
GAD65_504–518	VCFWYIPPSLRTLED	7.58	95.8	NB	25.2	10.3	Y
GAD65_511–525	PSLRTLEDNEERMSR	−0.89	NB	NB	NB	NB	Y
GAD65_550–570	LGDKVNFFRMVISNPAATHQD	22.32	—	<25	—	—	Y

**Table 2:** Treatment and control peptides for *in vitro* and *in vivo* studies. The following table contains information on the sequences, MHC promiscuity, and parental protein location of the peptides and controls used in these studies.

Peptide name	Amino acids	Sequence	HLA motifs	EpiMatrix cluster score	Location in IgG
hTregitope IgG-167	26	PAVLQSSGLYSLSSVVTVPSSSLGTQ	20	30.05	CH1
hTregitope IgG-289	21	EEQYNSTYRVVSVLTVLHQDW	14	22.57	CH2
mTregitope IgG-167	20	PAVLQSDLYTLSSSVTVPSS	n/a	n/a	CH1
mTregitope IgG-289	18	EEQFNSTFRSVSELPIMHQ	n/a	n/a	CH2

mPPI 7–23	17	FLPLLALLALWEPKPTQ	11	18.69	—
mPPI 20–35	16	KPTQAFVKQHLCGPHL	2	−2.82	—
mPPI 33–47	15	PHLVEALYLVCGERG	3	−0.69	—
mPPI 71–86	16	SPGDLQTLALEVARQK	0	−6.59	—
mPPI 77–92	16	TLALEVARQKRGIVDQ	2	−3.1	—

TetTox 830–844	15	QYIKANSKFIGITEL	13	25.65	—
Flu-HA 308–318	13	PRYVKQNTLKLAT	10	21.82	—
OVA 323–339	17	KISQAVHAAHAEINEAG	4	−0.39	—

**Table 3:** Two-step assay results. Diabetic and healthy subject PBMCs were incubated for 7 days with a pool of GAD65 epitopes with or without Tregitopes. The cells were then washed and put into ELISpot plates to test recall responses to individual epitopes, the same pool used in the primary culture, or whole GAD65 protein. ELISpot results were considered positive if the number of SFC over background per million cells was >50 (Bold). Spot counts that failed to meet this threshold were considered negative results (Italic). Due to variable PBMC yield and recovery after 7 days, not all epitopes were included in every ELISpot, as indicated by “ND”.

	Subject D1100H		Subject D1101H		Subject D1104H		Subject D1105H		Subject D1106H		Subject D1107H	
	Tregitope		Tregitope		Tregitope		Tregitope		Tregitope		Tregitope	
	−	+	−	+	−	+	−	+	−	+	−	+
GAD65_450–470	−*48 *	−*48 *	**260**	**324**	**77**	**69**	**73**	19*	**155**	24*	*22 *	−18 *
GAD65_550–570	**199**	**141**	**126**	**100**	**146**	**101**	**352**	228*	**88**	**113**	**160**	1**
GAD65_504–518	**101**	−66 *	*31 *	−*12 *	**72**	*11 *	**77**	*28 *	**82**	**74**	*7 *	−*18 *
GAD65_480–496	**51**	*39 *	**70**	22*	**129**	41*	**75**	*4*	**70**	**88**	*42 *	−*3 *
GAD65_511–525	**186**	−44 *	36	*32 *	*6 *	*26 *	*2 *	−*4 *	**54**	*23 *	*18 *	−*13 *
GAD65_264–287	**91**	*33 *	**61**	26*	**141**	**154**	**205**	138*	**173**	66*	*48*	−4 *
GAD65_369–392	**1121**	**1351**	**254**	**283**	**434**	266**	**468**	**406**	ND	ND	ND	ND
GAD65_355–372	**205**	**515**	**119**	49*	**151**	**268**	**488**	**509**	ND	ND	ND	ND
GAD65_203–226	ND	ND	**157**	**51**	**221**	**154**	**245**	138*	**207**	**138**	ND	ND
GAD65_243–267	ND	ND	**146**	35**	**71**	**54**	*30 *	**274**	**134**	**144**	ND	ND
GAD65_405–424	ND	ND	**71**	**56**	**126**	**64**	ND	ND	**324**	**365**	ND	ND
GAD65_086–103	**149**	**106**	**69**	*38 *	*21 *	*1 *	−*32 *	**76**	**59**	*46 *	*13 *	−*19 *
GAD65_338–354	ND	ND	**76**	*44 *	**54**	−4 *	ND	ND	*48 *	*24 *	ND	ND
GAD65_175–190	ND	ND	*40 *	*23 *	**72**	**59**	ND	ND	*33 *	*17 *	ND	ND
GAD65 protein	**413**	**77**	*37 *	**59**	*32 *	**69**	ND	ND	**670**	**591**	**95**	*46 *
GAD65 peptide pool	**1681**	**1429**	**403**	**259**	**391**	**378**	**645**	**579**	**552**	**543**	**262**	9**

	Negative Control 669		Negative Control 668		Negative Control 674		Negative Control 670		Negative Control 672			
	Tregitope		Tregitope		Tregitope		Tregitope		Tregitope			
	−	+	−	+	−	+	−	+	−	+		

GAD65_450–470	*33 *	*13 *	**125**	−10 **	**396**	**464**	**115**	23*	−*98 *	**63**		
GAD65_550–570	**144**	30**	**312**	**440**	**490**	**431**	*25 *	−*90 *	ND	ND		
GAD65_504–518	*24 *	*27 *	**222**	75*	*21 *	**76**	*39 *	−*26 *	*0 *	**133**		
GAD65_480–496	**313**	98**	**195**	**193**	**162**	69**	*−28 *	−*81 *	*8 *	*49 *		
GAD65_511–525	*13 *	−17 *	**138**	**103**	*19 *	*24 *	*36 *	−*79 *	**52**	**113**		
GAD65_264–287	*26 *	*12 *	**813**	**593**	**183**	**153**	**129**	*19 *	*5 *	*39 *		
GAD65_369–392	ND	ND	ND	ND	**750**	531*	ND	ND	ND	ND		
GAD65_355–372	ND	ND	ND	ND	**72**	17*	ND	ND	ND	ND		
GAD65_203–226	**109**	**52**	**475**	**460**	**168**	**109**	**329**	82**	ND	ND		
GAD65_243–267	*21 *	−*12 *	**287**	207*	**161**	**134**	**188**	−9 **	**67**	**59**		
GAD65_405–424	**78**	13**	**178**	−8 **	*16 *	*26 *	*49 *	−*14 *	−*10 *	*11 *		
GAD65_086–103	*11 *	*5 *	**143**	**128**	**51**	*34 *	*27 *	−*9 *	−*27 *	*16 *		
GAD65_338–354	ND	ND	ND	ND	*4 *	*14 *	ND	ND	ND	ND		
GAD65_175–190	*39 *	−*3 *	ND	ND	*11 *	*8 *	**53**	−7 *	ND	ND		
GAD65 protein	ND	ND	ND	ND	*44 *	**818**	ND	ND	ND	ND		
GAD65 peptide pool	**76**	*30 *	**820**	**692**	**613**	*42 *	**117**	−*19 *	**100**	*48 *		

≥**50 SFC over BG/10^6^**.

<*50 SFC over BG*/10^6^.

*P < 0.05.

**P < 0.01.

**Table 4:** Summary of responses of diabetic subjects to Tregitopes in vitro. Effects of Tregitopes on ELISpot responses to the panel GAD65 epitopes, as reported in detail in Table 3, are summarized here. In the two-step T cell assays, IFN*γ* ELISpot responses to GAD65 peptides were suppressed for 78% of the peptides tested using diabetic subject PBMC and for 74% of the peptides tested using normal control subjects. The suppression reached statistical significance for more than a quarter (26%) of all assays performed.

Subject ID	Number of peptides tested in ELISpot	Responses suppressed by Tregitopes	Responses suppressed significantly (*P* < 0.05)
D1100H	9	6 (67%)	2 (22%)
D1101H	14	12 (86%)	4 (29%)
D1104H	14	11 (79%)	3 (21%)
D1105H	11	8 (73%)	4 (36%)
D1106H	12	8 (67%)	2 (17%)
D1107H	7	7 (100%)	3 (43%)

N668C	10	9 (90%)	4 (40%)
N669C	11	10 (91%)	3 (27%)
N670C	11	11 (100%)	4 (36%)
N672C	8	1 (13%)	0 (0%)
N674C	14	9 (64%)	3 (21%)
